# Time course of functional and structural brain network changes after mild traumatic brain injury

**DOI:** 10.1093/braincomms/fcag072

**Published:** 2026-03-16

**Authors:** Eunkyung Kim, Han Gil Seo, Roh-Eul Yoo, Byung-Mo Oh

**Affiliations:** Department of Rehabilitation Medicine, Seoul National University Hospital, Seoul 03080, Republic of Korea; Biomedical Research Institute, Seoul National University Hospital, Seoul 03080, Republic of Korea; Department of Rehabilitation Medicine, Seoul National University Hospital, Seoul 03080, Republic of Korea; Department of Rehabilitation Medicine, Seoul National University College of Medicine, Seoul 03080, Republic of Korea; Department of Radiology, Seoul National University College of Medicine and Seoul National University Hospital, Seoul 03080, Republic of Korea; Department of Rehabilitation Medicine, Seoul National University Hospital, Seoul 03080, Republic of Korea; Department of Rehabilitation Medicine, Seoul National University College of Medicine, Seoul 03080, Republic of Korea; Institute on Aging, Seoul National University, Seoul 08826, Republic of Korea

**Keywords:** mild traumatic brain injury, attention networks, functional network connectivity, diffusion tensor imaging, longitudinal study

## Abstract

Traumatic brain injury is a progressive and potentially persistent pathophysiological condition affecting multiple cognitive domains. Large-scale brain networks, particularly those supporting attention, are closely linked to these cognitive impairments. Additionally, functional network connectivity, which captures statistical dependencies among network time courses, has revealed disrupted coupling between attentional networks. However, longitudinal evidence on how functional network connectivity changes over time and whether such changes are related to structural connectivity or cognitive outcomes remains limited. To address these gaps, this study investigated functional and structural connectivity among the default mode network, dorsal attention network, and ventral attention network in 41 patients with mild traumatic brain injury (mean age: 48.7 ± 15.8 years) and 35 matched controls (mean age: 44.6 ± 12.8 years). Both groups underwent brain imaging, clinical and neuropsychological assessments, and two cognitive tasks, including the Wisconsin Card Sorting Test and Digit Span Test, during the baseline (<1 month) and follow-up (>3 months) phases. Functional networks were defined using the Schaefer atlas and structural connectivity was constructed using these networks as nodes. Diffusion metrics, including fractional anisotropy, axial diffusivity, radial diffusivity, and mean diffusivity, were assessed. Clinically, symptom scores of depression, symptom severity, and quality of life improved over the three-month period (*P* < 0.001), whereas outcomes assessed by the Korean version of the Montreal Cognitive Assessment and the Frontal Assessment Battery showed limited change (*P* > 0.05). Cognitive performance was generally comparable between groups, except for a significant main effect of group in the backward Digit Span Test, with the mild traumatic brain injury group showing poorer performance than controls. In the baseline phase, functional network connectivity between the default mode network and dorsal and ventral attention network was significantly reduced in the mild traumatic brain injury group, correlating with impaired attentional control but not with working memory capacity. These disruptions were no longer observed at the follow-up assessment. Structural connectivity remained largely stable throughout the period. Diffusion metrics in controls were associated with attentional performance but not with working memory performance. Positive associations were observed between attentional performance and fractional anisotropy only during the follow-up phase in the mild traumatic brain injury group, possibly reflecting shifts in the association over time. In contrast, working memory performance was consistently linked to diffusion metrics in the mild traumatic brain injury group. Taken together, these findings highlight a temporal dissociation between early functional disconnection and later structure-cognitive coupling following mild traumatic brain injury and highlight the value of multimodal longitudinal imaging in understanding post-injury recovery.

## Teaser

Kim *et al*. report early (<1 month) functional disconnection between the default mode network and attention-related networks in mild traumatic brain injury, despite relatively stable structural connectivity. Functional network connectivity became comparable to controls at follow-up (>3 months), whereas fractional anisotropy became related to attentional performance.

## Graphical Abstract

**Graphical Abstract:**
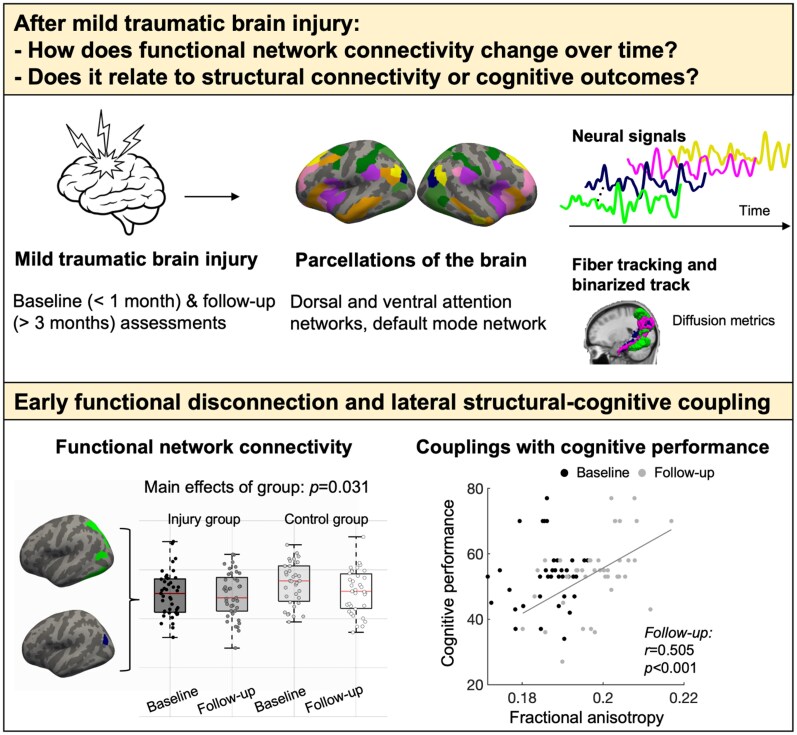

## Introduction

Cognitive impairment is one of the most consistently reported symptoms of traumatic brain injury (TBI), affecting multiple domains, including memory, language and sensory perception.^1,2^ These impairments are associated with alterations in attention-related brain networks, particularly the dorsal attention network (DAN) and ventral attention network (VAN),^3-5^ which support endogenous (goal-directed) and exogenous (stimulus-driven) attention, respectively.^6,7^ For example, heightened activation in DAN regions, including the right parietal and dorsolateral frontal areas, has been reported in response to increased working memory processing load,^3^ whereas hyperactivation in ventral frontoparietal regions (which are part of the VAN) has been observed during visuospatial attention tasks after TBI.^4^ Individuals with mild TBI (mTBI) exhibit poor attention performance when lesions are present within the DAN^8^ and have demonstrated reduced grey matter probability in cortical areas encompassing the DAN and VAN, observed from 6 weeks to 1 year post-injury.^9^

Although these findings highlight region-specific alterations within attention-related networks, recent studies have emphasized the importance of examining functional interactions between large-scale brain networks to comprehensively understand post-injury symptoms.^10-14^ Functional network connectivity (FNC), which captures statistical dependencies among network time courses, provides a framework for examining how distributed brain systems coordinate over time to produce different brain states.^15^ Several cross-sectional studies have examined FNC in individuals with mTBI. These studies have observed disrupted connectivity between the executive control network and the default mode network (DMN), which was significantly associated with attention performance during the acute phase.^10^ Moreover, studies report reduced FNC between the DMN and VAN in individuals with memory deficits within one month post-injury.^11^ Individuals with post-traumatic headache showed enhanced FNC between salience and executive control networks and reduced FNC between salience and sensorimotor networks.^12^ Post-concussive complaints were related to enhanced FNC between the bilateral frontal and salience networks during rest^13^ and enhanced FNC between the executive network and DMN during working memory tasks.^14^ Despite these findings, longitudinal evidence on how FNC changes over time and whether such changes are related to structural connectivity or cognitive outcomes remains limited, although TBI, including concussions, is a progressive and potentially persistent pathophysiological condition.^16,17^

To address these gaps, the present study investigated early and late changes in the FNC and structural connectivity among the DMN, DAN and VAN in individuals with mTBI and matched healthy controls in relation to cognitive performance. Participants underwent resting-state functional magnetic resonance imaging (rsfMRI) and diffusion tensor imaging (DTI) twice: within 1 month and at 3 months post-injury. Cognitive performance was assessed using card-sorting and digit-span tasks at both time points. DMN was selected for analysis because of its role in monitoring the external environment by dynamically modulating internally and externally oriented attention.^18^ DAN and VAN were included because they constitute the core attentional systems underlying goal-directed and stimulus-driven attention, respectively.^3-7^ By integrating functional, structural and behavioural data over time, this study aimed to identify how mTBI affects large-scale attention-related networks and how such changes relate to cognitive performance. In addition to examining large-scale network interactions, microstructural properties of the white matter pathways supporting these networks were assessed. Diffusion metrics, such as fractional anisotropy (FA), axial diffusivity (AD), radial diffusivity (RD), and mean diffusivity (MD), are sensitive to axonal or myelin-related alterations,^19^ providing complementary information about injury-related changes not captured by functional or structural connectivity alone. To the best of our knowledge, this is one of the first longitudinal investigations of attention-related network dynamics in patients with mTBI.

Based on prior evidence that post-concussive symptoms typically improve within 3 months post-injury,^20^ we hypothesized that individuals with mTBI would show early alterations in FNC, structural connectivity, and diffusion metrics that subsequently recover at follow-up. In particular, relationships with cognitive performance were expected to be positive for FA and negative for AD, RD, and MD during follow-up assessment, consistent with prior studies showing that higher FA and lower diffusivity values are associated with better performance in domains such as attention, executive function and global cognition.^21,22^ A multimodal approach was adopted to complement FNC, which reflects the dynamic coordination among large-scale networks, with measures of structural connectivity that index the anatomical pathways supporting these interactions, thereby providing a more comprehensive characterization of network-level disruption after injury. Mood, cognition and functional outcomes were assessed to aid interpretation of group-level and longitudinal differences, as these clinical measures offer complementary insight into longitudinal changes.

## Materials and methods

### Participants

All participants provided informed consent in accordance with the guidelines of Seoul National University Hospital. This consecutive, prospective follow-up study was conducted at a single centre (Seoul National University Hospital), where participants were recruited and underwent all MRI scans. This study was approved and monitored by the Institutional Review Board of Seoul National University Hospital (IRB Nos. 1804-047-936 and 2111-062-1271). Fifty-five individuals with mTBI who visited the outpatient clinic or emergency department between August 2018 and February 2023 were enrolled. The inclusion criteria for the mTBI were as follows: (i) mTBI diagnosis, (ii) age >20 years and (iii) availability of a brain imaging scan within 1 month after injury. Individuals were diagnosed with mTBI if they experienced a head impact associated with loss of consciousness (<30 min), post-traumatic amnesia (<24 h), focal neurological deficits or altered mental status. Healthy controls were recruited using posters placed on IRB-approved bulletin boards at the hospital. Fifty-three age-matched healthy individuals were included. No upper age limit was applied to either group. Age matching was achieved through stratification into 5-year age bands. The exclusion criteria for all participants included contraindications to MRI and pregnancy. Additional exclusion criteria for the mTBI group were (i) diagnosis of any other neurological or psychiatric disorder or (ii) use of medications for such conditions within 6 months before screening. Additional exclusion criteria for the control group included: (i) a history of mTBI, (ii) any other neurological or psychiatric disorders and (iii) a score of <23 on the Korean version of the Montreal Cognitive Assessment (K-MoCA), which is the cut-off for mild cognitive impairment.^23^ This criterion was applied only to healthy controls to exclude individuals with possible cognitive impairment. No additional restrictions were placed on past but fully resolved conditions. Incidental MRI findings were reviewed for all participants.

### Clinical assessments

Several clinical and neuropsychological assessments were conducted to evaluate the symptoms and functional status of individuals with mTBI, including the Beck Depression Inventory (BDI), K-MoCA,^23^ Frontal Assessment Battery (FAB),^24^ Glasgow Outcome Scale Extended (GOSE),^25^ Rivermead Post-Concussion Symptom Questionnaire (RPCSQ)^26^ and EuroQol 5-Dimension Questionnaire (EQ-5D).^27^ Higher scores on the BDI, RPCSQ and EQ-5D indicated greater symptom severity and functional impairment, whereas higher scores on the K-MoCA, FAB and GOSE reflected better cognitive function and overall recovery.

The Wisconsin Card Sorting Test (WCST) was administered as part of the Computerized NeuroCognitive Function Test (CNT40®; MaxMedica, Seoul, Korea)^28^ to assess executive functioning. This test provides several indices, including total errors, perseverative responses, perseverative errors and non-perseverative errors. Among these, non-perseverative errors are considered to reflect attentional control or inefficient cognitive monitoring, as they result from failures in maintaining attention.^29^ Total errors reflect general problem-solving abilities and overall executive functioning, whereas perseverative responses and errors indicate deficits in cognitive flexibility.^30^ The forward and backward Digit Span Tests (DTs)^31^ were used to assess working memory capacity. All cognitive outcome scores were converted to standardized *t*-scores (mean = 50, standard deviation = 10) based on age-matched normative samples, with higher scores indicating better cognitive performance.

Control participants completed the same assessments, except for the GOSE, RPCSQ and EQ-5D. For the mTBI group, all assessments were performed within 1 week of each MRI session, except for two participants who completed the WCST and DTs 12 and 22 days after the baseline MRI scan, and three who completed paper-based assessments 8 days before the baseline MRI. All controls completed assessments within 7 days of imaging.

### Brain imaging acquisition

Eligible participants underwent brain MRI within 1 month and at 3 months post-injury using a 3-T Magnetom Triotim scanner (Siemens, Erlangen, Germany). All scans were performed using identical acquisition parameters on the same 3-T MRI scanner throughout the study, and no scanner upgrades or protocol modifications occurred during data collection. The rsfMRI, DTI and structural T1-weighted images were obtained using the following parameters. An interleaved slice acquisition with 116 volumes was used for rsfMRI, with a voxel size of 1.9 × 1.9 × 3.5 mm, an image matrix of 128 × 128, repetition time (TR) of 3500 ms, echo time (TE) of 30 ms, field of view (FOV) of 240 mm^2^ and a flip angle of 90°. DTI data were acquired using single-shot spin-echo echo-planar imaging sequences, with a voxel size of 1.9 × 1.9 × 3 mm, an image matrix of 128 × 128, TR of 5800 ms, TE of 88 ms and FOV of 240 mm^2^. A total of 30 diffusion directions with a *b*-value of 1000 s/mm^2^ and one *b* = 0 image were obtained. A sagittal 3D Turbo-FLASH sequence was used for structural T1-weighted imaging, with isotropic 1 mm^3^ voxels, an image matrix of 256 × 256, TR of 1670 ms, TE of 1.89 ms, FOV of 250 mm^2^ and flip angle of 9°.

### FMRI preprocessing and constructing functional network connectivity

Image preprocessing was performed using the Functional Magnetic Resonance Imaging of the Brain (FMRIB) Software Library (FSL, version 6.0.1, http://www.fmrib.ox.ac.uk/fsl).^32^ Detailed preprocessing procedures for rsfMRI have been described in our previous study.^33^ After discarding the first four volumes of each scan, corrections for head motion and slice timing were applied. Additionally, head motion was quantified using framewise displacement (FD).^34^ The proportion of volumes exceeding the FD > 0.5 mm threshold was very small in both groups (controls: 0.5%; mTBI: 1.2%); therefore, no rsfMRI scans were excluded. The motion-corrected images were spatially normalized in two stages: the rsfMRI data were registered to the structural T1-weighted image using boundary-based registration,^35^ and the T1 image was normalized to the standard Montreal Neurological Institute space using linear and nonlinear transformations. Spatially normalized images were smoothed using a Gaussian kernel (sigma = 2.55) to enhance the signal-to-noise ratio. To reduce motion-related artefacts, we applied Independent Component Analysis–Automatic Removal of Motion Artefacts,^36^ an automated method that identifies and removes motion-related independent components. Signals from white matter and cerebrospinal fluid were regressed out, and the blood-oxygen-level-dependent signal was band-pass filtered to retain frequencies within the range of 0.01 *<*  *f* < 0.1 Hz for further analysis.

**Figure 1:**
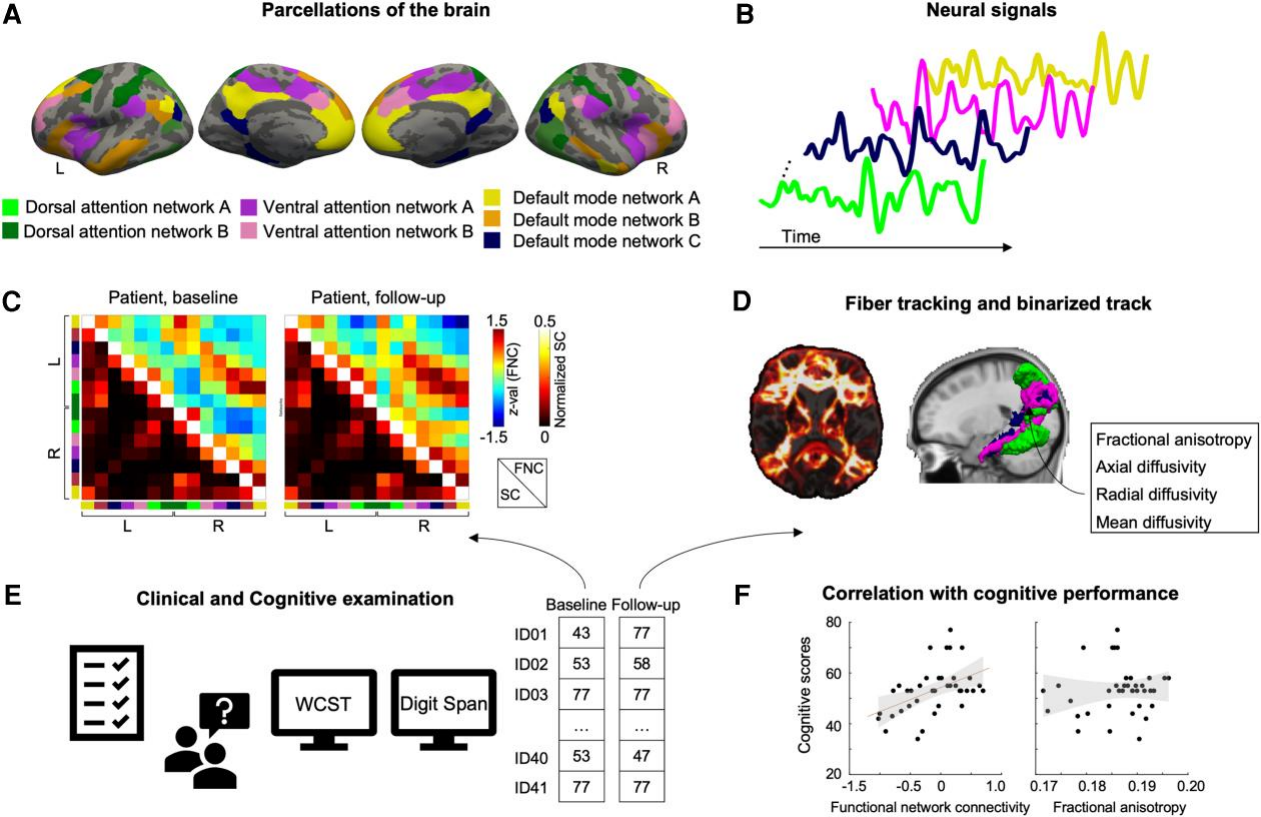
**Schematic representation of the overall study design and analysis pipeline.** (**A**) Brain parcellation based on functionally defined networks from the Schaefer atlas. (**B**) Extraction of neural time series from each network. (**C**) Construction of FNC and SC at two-time points (baseline and follow-up) for each participant. (**D**) SC analysis, including tractography and extraction of diffusion metrics such as FA, AD, RD and MD. (**E**) Clinical and cognitive assessments at two-time points (baseline and follow-up), including the WCST and Digit Span Task. (**F**) Correlation analyses between cognitive scores and imaging-derived connectivity metrics, such as FNC or FA.

A previously reported Schaefer functional atlas was employed to construct the FNC among the DAN, VAN and DMN.^37,38^ The Schaefer atlas segments the brain into parcels, with *L* representing the number of parcels (*L* = 400, 600, 800 or 1000), using a gradient-weighted Markov Random Field model that integrates both local gradient and global similarity approaches. The segmented areas were clustered into 17 functionally homogeneous networks (17 networks, 400 parcels, 2-mm resolution). Two subdivisions of DAN (DAN-A and DAN-B), two of VAN (VAN-A and VAN-B) and three of DMN (DMN-A, DMN-B and DMN-C) were included in the 17 networks in each hemisphere (Fig. 1A). The Schaefer parcels assigned to each subnetwork and their anatomical regions are summarized in Supplementary Table 1.

The time-series data of all voxels were averaged for each network (Fig. 1B), and Pearson’s correlation coefficients were calculated between pairs of networks to construct the FNC.^39^ The resulting correlation coefficient (*r-*values*)* were transformed into *z-*scores using Fisher’s *r*-to-*z* transformation to approximate a normal distribution and enable subsequent statistical analysis. An example FNC matrix for an individual with mTBI is shown in the upper triangular matrix in Fig. 1C.

### DTI preprocessing and constructing structural connectivity

Eddy current distortion and motion artefacts in the DTI images were corrected using the FMRIB’s Diffusion Toolbox.^40^ After eddy current correction, head motion was quantified from the rigid-body realignment parameters estimated by MCFLIRT. Motion was summarized using translation-based displacement measures (absolute and inter-volume displacement).^41^ All displacement values in the present dataset were small (absolute: ≤ 0.342 mm; inter-volume: ≤ 0.209 mm across all participants) and remained below levels previously shown to affect diffusion estimates.^41^ Accordingly, no DTI data were excluded based on motion. Non-brain tissue was removed from the *b*0 image using the Brain Extraction Tool.^42^ The distribution of fibre tract orientations at each voxel was estimated using Bedpostx, implemented in FSL, based on Markov Chain Monte Carlo sampling.^43^ The estimated probability distribution of fibre directions at each voxel was used to trace white matter fibre tracts between node pairs. The predefined DAN, VAN and DMN regions served as nodes to construct structural connectivity. Fibre tracking was initiated within each region using 5000 sample streamlines per voxel (Fig. 1D). The tracking parameters were as follows: number of steps per sample = 2000, step length = 0.5 mm, and curvature threshold = 0.2. Modified Euler integration was used to compute the tractography. This process generated a structural connectivity matrix, which was subsequently normalized to account for individual variability in brain size by dividing the total number of streamlines originally generated from the seed node (the number of voxels within the node × 5000). The normalized streamlines from node *i* to node *j* and from node *j* to node *i* were averaged to define unidirectional structural connectivity.^44^ An example of one individual is visualized as the lower triangular elements in the matrix of Fig. 1C.

A streamlined density map was generated for each individual using the same tracking parameters; however, simple Euler integration was used to reduce computational time. The density map reflects the number of streamlines reaching each voxel. The average number of originally generated streamlines between pairs of nodes was used to normalize the density map, as recorded in the Waytotal file. The normalized density map was spatially transformed to MNI space using a concatenated transformation derived from a two-stage registration: the *b*0 image was first registered to the structural T1 image using nonlinear registration, followed by transformation of the T1 image to template space using nonlinear registration. The total number of streamlines with a >10% probability in tractography was used to represent the structural connection between two nodes. This was binarized for each individual by excluding streamlines with <10% probabilities and retaining those with >90% (Fig. 1D). The union of the binarized tracts from both groups at each time point served as a mask to extract diffusion metrics, including FA, AD, RD and MD. These diffusion metrics were estimated for each voxel using a tract-based spatial statistical approach. The diffusion tensor matrix was used to compute three eigenvalues during this process: *λ*_1_, *λ*_2_ and *λ*_3_. AD was defined as the diffusivity parallel to the axon (*λ*_1_), RD was defined as the diffusivity perpendicular to the axon ((*λ*_2_ + *λ*_3_)/2), whereas MD represented the average diffusivity across all three eigenvalues ((*λ*_1_ + *λ*_2_ + *λ*_3_)/3). FA represented the normalized variance of the three eigenvalues. Individual FA, AD, RD and MD maps were nonlinearly registered to the standard space and projected onto a mean FA skeleton image to enhance the analysis.^45^

### Statistical analysis

#### Comparing clinical assessments

Normality of continuous variables was assessed using the Shapiro–Wilk test (*P* < 0.05). Group differences in age and sex were assessed using an independent-sample *t*-test and the chi-squared test, respectively, as age was normally distributed. Clinical characteristics, including the BDI, FAB, K-MoCA, GOSE, RPCSQ and EQ-5D, were compared cross-sectionally and longitudinally using the Wilcoxon rank-sum and Wilcoxon signed-rank tests, respectively. Statistical significance was set at *P* < 0.05.

Sub-scores from the WCST and DTs were analyzed using a linear mixed-effects model including group, time and their interaction as fixed effects; participants as a random effect; and age and sex as covariates. Multiple comparisons were controlled using the false discovery rate (FDR) procedure,^46^ with significance defined as FDR-adjusted *P* < 0.05. FDR correction was applied only to the cognitive function test scores. Post-hoc analyses were conducted using two-sided unpaired or paired-sample *t*-tests (uncorrected *P* < 0.05). Three participants in the mTBI group did not complete the WCST or DTs and were excluded from the cognitive analyses.

Potential outliers were evaluated using a statistical threshold of ±3 standard deviations. No outliers were present in the control group. Isolated outliers were observed for K-MoCA, FAB and EQ-5D in the patient group, whereas no WCST or DTs sub-scores contained outliers. All values were retained because they remained clinically plausible, and non-parametric tests were used owing to violations of normality assumptions.

#### Functional network connectivity and structural connectivity analyses

A linear mixed-effects model was used to analyze both FNC and structural connectivity, including group, time and their interaction as fixed effects; participants as a random effect; and age and sex as covariates. Multiple comparisons were corrected using the FDR approach to ensure statistically conservative findings.^46^ Considering the subtle nature of mTBI and the relatively small sample size, uncorrected results at *P* < 0.05 were also reported. Post-hoc analyses following the main effects were conducted using two-sided unpaired or paired-sample *t*-tests.

#### Relationship between cognitive outcomes and connectivity metrics from fMRI and diffusion imaging

Linear mixed-effects models including an interaction term between FNC (or structural connectivity features) and time were conducted to examine whether the relationship between cognitive performance and FNC or structural connectivity features changed over time in the mTBI group. The individual was modeled as a random intercept to account for within-individual repeated measures. Only connectivity pairs showing significant group effects in the prior mixed-effects analyses were tested. Cognitive performance was indexed by non-perseverative error scores from the WCST and backward digit-span scores from the DT. These cognitive measures were selected to evaluate the association between attention-related internetwork connectivity and domains of cognitive function, including attentional control and working memory capacity. Simple Pearson correlations were evaluated between selected cognitive measures and FNC or structural connectivity features at the baseline and follow-up assessments to aid interpretation and visualize the direction and strength of these relationships at each time point (Fig. 1E and F). Associations between diffusion metrics, including FA, AD, RD and MD, and cognitive performance were also evaluated using the same analytic framework. Pearson correlations between diffusion metrics and cognitive scores were computed separately for each group and each time point for descriptive purposes.

Supplementary linear mixed-effects models incorporating the three-way interaction term (feature × time × group) were conducted to formally test whether the strength of these feature (FNC, structural connectivity and diffusion metrics)—cognition relationships differed between groups or changed over time. These models were applied to only those connections or pathways showing significant effects in the corresponding primary analyses.

## Results

### Demographic data

Among the 55 individuals with mTBI initially enrolled, 41 (mean age 48.7 ± 15.8 years at the time of first brain imaging; 22 female) were included in the analyses. Information regarding participants lost to follow-up is provided in the Supplementary Results. Participants underwent their first MRI scan within 1 month post-injury (mean 20.0 ± 6.3 days; range 8–30 days) and a second scan approximately 3 months post-injury (mean 102.5 ± 17.4 days; range 90–177 days), with an average interval of 82.4 ± 17.8 days between scans. Clinical assessments were completed within 1 week of each imaging session. In the control group, 53 participants were initially enrolled, of whom 35 controls (mean age 44.6 ± 12.8 years at first imaging) completed two MRI scans with an interval similar to their matched patients (mean 81.1 ± 13.2 days). The interval between scans was generally consistent, with a difference of approximately 2 weeks for most matched pairs. However, one participant had a longer interval (15 days) due to scheduling difficulties, and another had a shorter interval (66 days) due to a temporary loss of contact with their matched patient. Six patients aged 60, 66, 71, 76 and 84 years did not have matching controls.

### Clinical assessments

Clinical assessment outcomes were compared both within and between groups (Table 1). No significant differences were observed in age or sex between the groups (*P* = 0.230 and *P* = 0.761, respectively). BDI, GOSE, RPCSQ and EQ-5D scores significantly improved in individuals with mTBI after the 3-month follow-up, indicating symptom recovery (*P* < 0.001). However, K-MoCA and FAB scores showed no significant difference between initial and follow-up assessments (*P* = 0.702 and *P* = 0.197, respectively). No significant changes were observed in BDI, K-MoCA or FAB scores between initial and follow-up assessments in the control group.

**Table 1:** Comparisons of clinical assessment outcomes both within and between groups

	mTBI			Controls			mTBI versus controls	
	Baseline	Follow-up	*P*-value	Baseline	Follow-up	*P*-value	Baseline (*P*-value)	Follow-up (*P*-value)
*Demographics*								
Age	48.66 (15.75)			44.63 (12.82)			0.230	
Sex	22/19 (F/M)			20/15 (F/M)			0.761	
*Cognitive and clinical outcome measures*								
BDI	13.68 (11.08)	9.12 (10.15)	<0.001	3.77 (3.86)	3.94 (3.21)	0.674	<0.001	0.085
K-MoCA a	25.12 (3.73)	25.30 (3.07)	0.702	27.09 (1.99)	27.54 (1.79)	0.136	0.015	<0.001
FAB b	16.24 (1.89)	16.43 (1.88)	0.197	17.31 (1.13)	17.34 (1.00)	0.811	<0.001	0.003
GOSE	6.02 (1.04)	6.88 (0.98)	<0.001					
RPCSQ	22.59 (16.02)	16.56 (13.79)	<0.001					
EQ-5D	7.51 (1.80)	6.51 (1.72)	<0.001					

*Abbreviations:* mTBI, mild traumatic brain injury; BDI, Beck Depression Inventory; K-MoCA, Korean version of the Montreal Cognitive Assessment; FAB, Frontal Assessment Battery; GOSE, Glasgow Outcome Scale Extended; RPCSQ, Rivermead Post-Concussion Symptoms Questionnaire; EQ-5D, EuroQol 5-Dimension Questionnaire; F/M, female/male.

^a,b^One individual with mTBI had missing follow-up data for the K-MoCA and FAB. The average and standard deviation of the follow-up data were calculated excluding this individual. Statistical tests were performed without including this participant.

Between-group comparisons revealed significant differences in BDI, K-MoCA and FAB scores at both initial and follow-up assessments, indicating prolonged symptoms in individuals with mTBI. The exception was the BDI comparison at follow-up, which showed marginal significance (*P* = 0.085).

### Cognitive performance

The results of the linear mixed-effects models for the WCST and DTs are summarized in Table 2, reporting unadjusted and covariate-adjusted *P*-values. After correction for multiple comparisons, only the forward and backward DTs showed significant group effects based on the unadjusted *P*-values. Although the covariate-adjusted model for the backward DT revealed a significant group effect, this result did not remain significant after correction for multiple comparisons.

**Table 2:** Results of linear mixed-effect models for computerized neurocognitive function test performance in individuals with mild traumatic brain injury and healthy controls

Subtest	Main effect of group		Main effect of time	
	Unadjusted *P*-value	Adjusted *P*-value a	Unadjusted *P*-value	Adjusted *P*-value
Card sorting b				
Total error	0.373	0.405	0.826	0.552
Perseverative response	0.891	0.813	0.487	0.719
Perseverative errors	0.730	0.866	0.422	0.517
Non-perseverative errors	0.267	0.356	0.975	0.521
Digit span				
Forward	0.019*	0.200	0.223	0.154
Backward	0.001*	0.040	0.386	0.165

^a^Adjusted models included age, sex, time and group-by-time interaction as covariates.

^b^Three individuals with mild traumatic brain injury had missing initial data for the WCST and were excluded from the statistical analysis for this subtest.

^*^Multiple comparisons were corrected using the false discovery rate method.

Post-hoc analyses indicated that the significant group effects in both the forward and backward DTs were driven by poorer performance in the mTBI group at both the initial (*P* = 0.086 and *P* = 0.015, respectively) and follow-up assessments (*P* = 0.088 and *P* = 0.023, respectively) than in the controls (Supplementary Table 2).

### Changes in functional network connectivity

**Figure 2:**
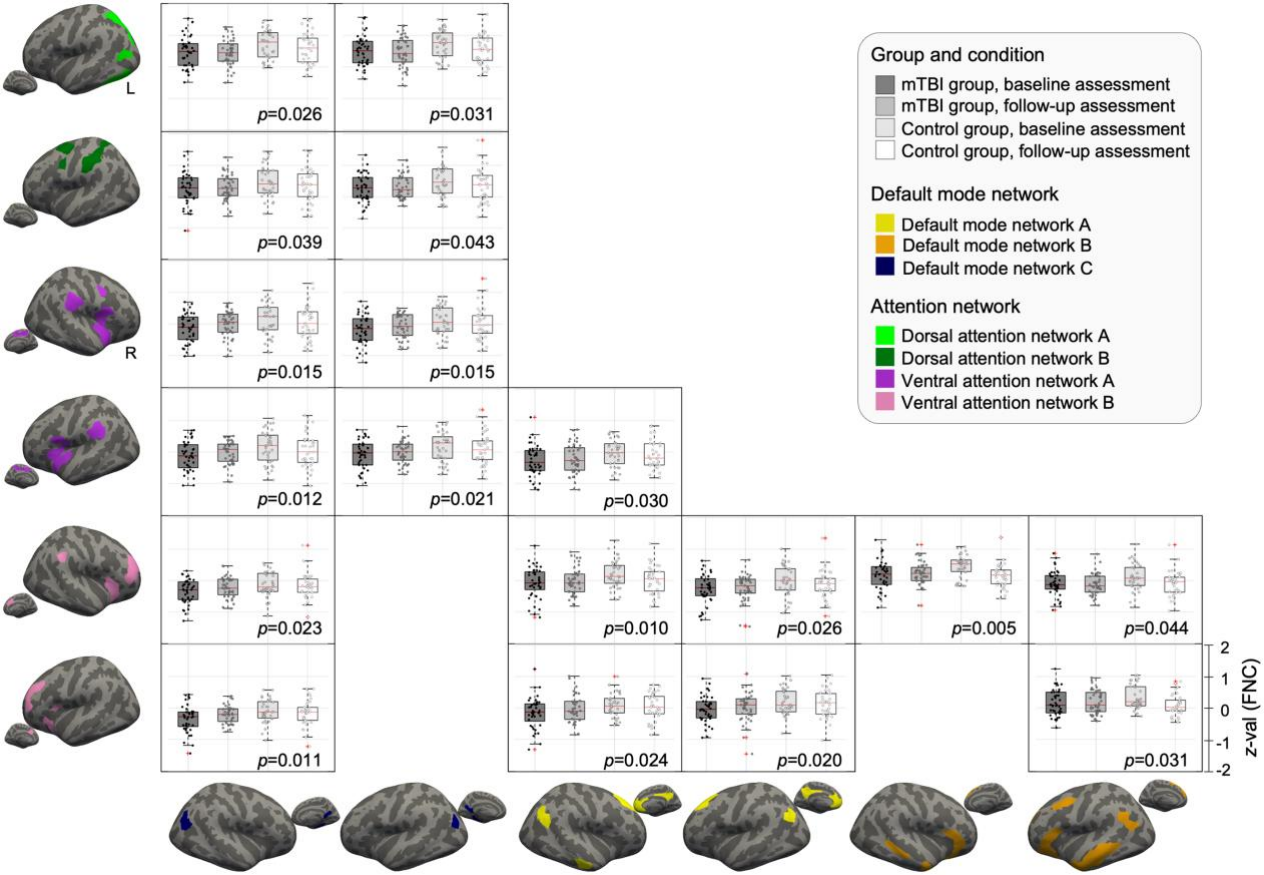
**Functional network connectivity (FNC) showing significant group effects.** Linear mixed-effects models identified 18 FNC pairs with significant main effects of group (*P* < 0.05; test statistics are provided in Table 3). Brain surface maps illustrate the cortical regions involved in each significant FNC pair, coloured according to network identity (see legend). Boxplots show *z*-transformed FNC values across four conditions: mild traumatic brain injury (mTBI, *n* = 41) and control groups (*n* = 35) at baseline and follow-up assessments. Each data point represents the *z*-value of FNC from one participant at each time point in each group. The *x*-axis indicates the four experimental conditions (mTBI baseline, mTBI follow-up, control baseline and control follow-up), while the *y*-axis represents *z*-transformed FNC values. Significant differences were observed in connectivity between the default mode network and attention networks (dorsal and ventral). Colours correspond to network parcellations from the Schaefer atlas: yellow (DMN-A), orange (DMN-B), navy (DMN-C), light green (DAN-A), dark green (DAN-B), purple (VAN-A) and pink (VAN-B).

After multiple comparison corrections, linear mixed-effects models revealed a significant main effect of time only for the connection between the right DMN-B and the right VAN-B, with none of the group effects remaining significant. In the uncorrected analyses, significant group effects were observed in 18 FNC pairs (Fig. 2, Table 3, Supplementary Results 2), particularly involving connections between DMN and either VAN or DAN. Post-hoc analyses indicated that FNC was lower in the mTBI group than in the control group at the initial time point, with no significant group differences observed at follow-up. Significant main effects of time were observed in six FNC pairs (Table 3). Temporal changes in several connections were observed in the control group, whereas no significant longitudinal changes were detected in the mTBI group.

**Table 3:** Significant findings of linear mixed-effects analysis comparing functional network connectivity in individuals with mild traumatic brain injury and healthy controls

Functional network connectivity		Main effects (linear mixed-effects)				Post-hoc comparisons (*P*-value)			
		Group		Time		mTBI versus Controls		Baseline versus Follow-up	
		*β*	*P*-value	*β*	*P*-value	Baseline	Follow-up	mTBI	Controls
L. DMN-A	L. VAN-B	−0.229	0.020	−0.053	0.576	0.018	0.351	0.365	0.592
	R. VAN-B	−0.229	0.026	−0.113	0.193	0.025	0.271	0.790	0.186
L. DMN-B	R. DMN-B	0.121	0.200	0.272	0.002	0.255	0.015	0.370	0.000
	L. VAN-B	−0.184	0.031	−0.242	0.002	0.044	0.245	0.619	0.005
	R. VAN-B	−0.182	0.044	−0.186	0.012	0.050	0.762	0.745	0.020
L. DMN-C	L. VAN-A	−0.231	0.021	−0.062	0.467	0.021	0.332	0.269	0.450
	R. VAN-A	−0.239	0.015	−0.076	0.352	0.012	0.516	0.102	0.376
	L. DAN-A	−0.223	0.031	−0.167	0.095	0.036	0.521	0.868	0.071
	L. DAN-B	−0.213	0.043	−0.126	0.203	0.045	0.689	0.571	0.239
R. DMN-A	L. VAN-A	−0.231	0.030	−0.050	0.568	0.025	0.149	0.722	0.513
	L. VAN-B	−0.223	0.024	−0.042	0.651	0.031	0.473	0.235	0.631
	R. VAN-B	−0.264	0.010	−0.186	0.019	0.012	0.560	0.673	0.017
R. DMN-B	L. VAN-B	−0.147	0.125	−0.214	0.017	0.153	0.484	0.986	0.014
	R. VAN-B	−0.254	0.005	−0.308	<0.001 a	0.007	0.369	0.652	0.001
R. DMN-C	L. VAN-A	−0.270	0.012	−0.110	0.193	0.014	0.495	0.164	0.235
	L. VAN-B	−0.214	0.011	−0.060	0.459	0.013	0.589	0.085	0.474
	R. VAN-A	−0.257	0.015	−0.083	0.347	0.013	0.590	0.078	0.403
	R. VAN-B	−0.191	0.023	−0.045	0.580	0.021	0.594	0.102	0.610
	L. DAN-A	−0.237	0.026	−0.121	0.224	0.034	0.235	0.933	0.216
	L. DAN-B	−0.233	0.039	−0.112	0.243	0.044	0.440	0.600	0.293

^a^Multiple comparisons were corrected using the false discovery rate method.

*Abbreviations:* L, left; R, right; DMN-A, default mode network A; DMN-B, default mode network B; DMN-C, default mode network C; VAN-A, ventral attention network A; VAN-B, ventral attention network B; DAN-A, dorsal attention network A; DAN-B, dorsal attention network B.

### Relationship between cognitive performance and functional network connectivity

**Figure 3:**
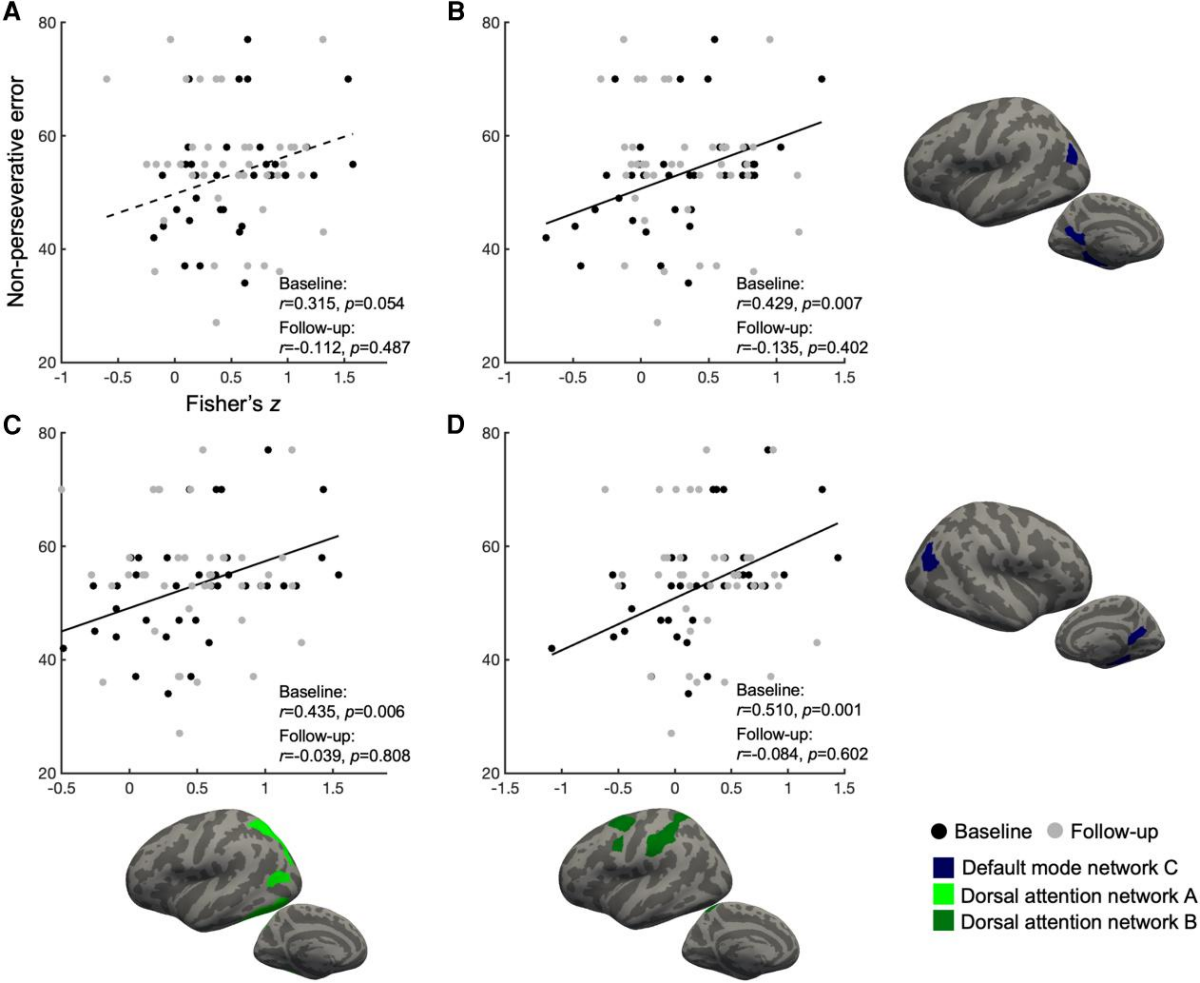
**Associations between functional network connectivity and cognitive performance in the mTBI group at baseline.** Cortical renderings (bottom row and right column) highlight regions involved in each functional network connectivity (FNC) pair, colour-coded by network: default mode network C (navy), dorsal attention network A (light green), and dorsal attention network B (dark green), based on the Schaefer atlas. Scatter plots (A–D) show positive associations assessed by Pearson’s correlation coefficient between *z*-transformed FNC values and non-perseverative error scores on the WCST, a measure of attentional control. Each data point represents one participant. Black dots indicate participants in the mTBI group at baseline (*n* = 41), while grey dots indicate the same participant at follow-up (*n* = 41). The *x*-axis represents the *z*-transformed FNC values between: (**A**) left DMN-C and left dorsal attention network A, (**B**) left DMN-C and left DAN-B, (**C**) right DMN-C and left DAN-A and (**D**) right DMN-C and left DAN-B. The *y*-axis represents non-perseverative error scores from the WCST.

Follow-up linear mixed-effects models were conducted in the mTBI group using the FNC pairs identified in a prior step, showing main effects of group, to investigate whether the association between FNC and cognitive performance changed over time. Pairs exhibiting significant main effects of time were excluded, as these effects were primarily driven by changes in the control group. In these follow-up models within the mTBI group, the main effects of FNC and time are summarized in Table 4 and are not the primary focus of this section. No interaction effects remained significant after correcting for multiple comparisons. Nevertheless, uncorrected results revealed several notable patterns. Regarding non-perseverative error scores, significant FNC × time interactions were observed primarily in connections between DMN-C and DAN. Specifically, the left DMN-C showed time-dependent associations with left DAN-A (*P* = 0.049) and DAN-B (*P* = 0.012), and right DMN-C showed a similar effect with left DAN-B (*P* = 0.014), with a trend-level effect for its connection with left DAN-A (*P* = 0.073). An additional three-way linear mixed-effects analysis revealed significant interaction effects of group × FNC × time for the connection between the left DMN-C and DAN-B (*P* = 0.028), along with a marginally significant group × time interaction (*P* = 0.051). In contrast, no significant associations were observed for backward digit-span scores, except for one FNC pair showing a significant interaction effect (left DAN-B and right VAN-B, *P* = 0.035), without corresponding main effects of FNC or time. Scatter plots of the simple correlations were provided to visualize the direction and magnitude of these FNC–cognition relationship at each time point (Fig. 3, Table 4).

**Table 4:** Time-dependent associations between functional network connectivity and non-perseverative error scores on the WCST in the mTBI group

Functional network connectivity		FNC × time interaction a		Main effects		Cross-sectional correlations (mTBI) b	
		*β*	*P*-value	FNC (*β*, *P*)	Time (*β*, *P*)	Baseline *r (P)*	Follow-up *r (P)*
L. DMN-C	L. DAN-A	−9.628	0.049	6.767 (0.054)	6.344 (0.041)	0.315 (0.054)	−0.112 (0.487)
	L. DAN-B	−12.676	0.012	8.367 (0.012)	5.285 (0.028)	0.429 (0.007)	−0.135 (0.402)
R. DMN-C	L. DAN-A	−8.489	0.073	7.124 (0.024)	5.544 (0.062)	0.435 (0.006)	−0.039 (0.808)
	L. DAN-B	−10.708	0.014	8.484 (0.004)	4.244 (0.049)	0.510 (0.001)	−0.084 (0.602)
L. DMN-B	R. VAN-B	−10.583	0.035	5.866 (0.111)	0.636 (0.692)	−0.002 (0.992)	0.154 (0.337)
L. DMN-C	L. VAN-A	4.619	0.375	6.782 (0.036)	0.663 (0.730)	0.378 (0.019)	0.398 (0.010)
	R. VAN-A	0.946	0.847	8.614 (0.009)	0.271 (0.893)	0.463 (0.003)	0.355 (0.023)
R. DMN-A	L. VAN-A	−1.944	0.651	6.784 (0.035)	0.812 (0.701)	0.364 (0.025)	0.205 (0.199)
	L. VAN-B	−0.155	0.973	6.592 (0.043)	0.840 (0.644)	0.300 (0.068)	0.249 (0.116)
R. DMN-C	L. VAN-A	−1.559	0.756	7.727 (0.018)	0.551 (0.771)	0.407 (0.011)	0.237 (0.135)
	R. VAN-A	−2.937	0.544	8.710 (0.010)	0.204 (0.916)	0.474 (0.003)	0.232 (0.145)

*Abbreviations*: WCST, Wisconsin Card Sorting Test; mTBI, mild traumatic brain injury; L., left; R., right; DMN-B, default mode network B; DMN-C, default mode network C; DAN-A, dorsal attention network A; DAN-B, dorsal attention network B; VAN-A, ventral attention network A; VAN-B, ventral attention network B.

^a^Linear mixed-effects models were conducted within the mTBI group to examine time-dependent associations between functional network connectivity and non-perseverative error scores on the WCST, including the main effects of functional network connectivity and time, as well as their interaction.

^b^Cross-sectional Pearson’s correlation analyses at baseline and follow-up were additionally performed to facilitate interpretation of the direction and magnitude of these associations.

### Changes in structural connectivity

Linear mixed-effects models revealed significant group effects for structural connectivity between left DAN-B and both left DAN-A and right VAN-A. The analysis revealed significant main effects of time between left DAN-A and both left DMN-B and right VAN-A (Supplementary Table 3). However, none of these effects remained significant after correction for multiple comparisons.

Post-hoc analyses indicated that individuals with mTBI showed marginally reduced structural connectivity between left DAN-A and DAN-B at the initial assessment and decreased connectivity between left DAN-B and right VAN-A at the initial and follow-up assessments. Among the connections that showed significant main effects of time, the change between left DAN-A and right VAN-A was primarily driven by increased connectivity in the control group at the follow-up assessment.

### Correlations between cognitive performance and structural connectivity

Neither non-perseverative error scores for WCST nor backward digit-span scores showed significant associations with group, time or their interaction in the mixed-effects models when examining DTI-based structural connectivity. Consistent with these results, none of the structurally altered connections demonstrated significant correlation with this cognitive performance.

### Relationship between cognitive performance and diffusion metrics for white matter pathways

Linear mixed-effects models were conducted using FA, AD, RD and MD as predictors of cognitive scores to examine whether diffusion characteristics of white matter pathways were associated with cognitive performance, applying the same set of connections used in the FNC analyses. After correction for multiple comparisons, none of the diffusion metrics showed significant associations with backward digit-span scores. In contrast, regarding non-perseverative error scores, several pathways demonstrated significant main effects of FA, and all pathways showed significant main effects of AD, RD and MD, indicating consistent associations between diffusion properties and task performance. However, neither the main effect of time nor the diffusion metrics × time interaction reached significance for any metric, suggesting that these relationships did not significantly differ between the initial and follow-up assessments (Supplementary Table 4). Nevertheless, several uncorrected diffusion metrics × time interactions were observed for connections between left DAN-B and bilateral DMN-C across FA, RD, and MD. An additional three-way linear mixed-effects analysis identified group × FA × time interactions for these connections (*P* = 0.025 for left DAN-B and DMN-C, *P* = 0.038 for the left DAN-B and right DMN-C), along with significant group × time interactions (*P* = 0.028 for left DAN-B and DMN-C, *P* = 0.040 for the left DAN-B and right DMN-C).

Pearson correlations stratified by group and time point are provided in Table 5 for non-perseverative error scores. Although the mixed-effects models did not yield significant diffusion metrics × time interactions, the correlations showed numerical differences across time within each group. For example, the mTBI group showed significantly positive FA–WCST correlations at follow-up but not at baseline, whereas controls showed relatively stronger positive correlations at the initial assessment. Regarding backward digit-span scores, the mTBI group showed similar descriptive patterns to those observed for non-perseverative error scores, whereas controls did not show meaningful correlations at either time point.

**Table 5:** Pearson correlation values between diffusion metrics and WCST non-perseverative error scores, stratified by group and time point

Group	Time	Diffusion metric	*r*-value (median, range)	*P*-value (median, range)
mTBI	Baseline	FA	0.220 (0.095–0.322)	0.186 (0.048–­0.569)
		AD	−0.446 (−0.499–−0.403)	0.005 (0.001–0.012)
		RD	−0.434 (−0.496–−0.387)	0.007 (0.002­–0.016)
		MD	−0.439 (−0.499–−0.396)	0.006 (0.001–0.014)
	Follow-up	FA	0.545 (0.499–0.585)	<0.001
		AD	−0.475 (−0.567–−0.431)	0.002 (<0.001–0.005)
		RD	−0.541 (−0.644–−0.512)	<0.001
		MD	−0.517 (−0.621–−0.489)	<0.001 (<0.001–0.001)
Controls	Baseline	FA	0.513 (0.418–0.576)	0.002 (<0.001–0.012)
		AD	−0.525 (−0.632–−0.330)	0.001 (<0.001–0.053)
		RD	−0.602 (−0.700–−0.509)	<0.001 (<0.001–0.002)
		MD	−0.582 (−0.689–−0.456)	<0.001 (<0.001–0.006)
	Follow-up	FA	0.320 (0.285–0.353)	0.061 (0.038–0.097)
		AD	−0.493 (−0.539–−0.433)	0.003 (<0.001–0.009)
		RD	−0.559 (−0.614–−0.512)	<0.001 (<0.001–0.002)
		MD	−0.547 (−0.592–−0.490)	<0.001 (<0.001–0.003)

Values (*r*- and *P*-) represent median Pearson correlation coefficients across all 18 network connections showing significant group-level functional network connectivity differences (uncorrected); ranges indicate minimum–maximum values across connections. Correlations are provided for descriptive purposes.

*Abbreviations*: WCST, Wisconsin Card Sorting Test; mTBI, mild traumatic brain injury; FA, fractional anisotropy; AD, axial diffusivity; RD, radial diffusivity; MD, mean diffusivity.

## Discussion

This study provides the first evidence of longitudinal changes in attention-related network connectivity following mTBI using a multimodal approach that integrates functional, structural and cognitive performance. Individuals with mTBI demonstrated reduced FNC, particularly between DMN and VAN, during the baseline assessment (<1 month), although these findings should be interpreted cautiously because most effects did not remain significant after correction for multiple comparisons. This reduction was significantly associated with non-perseverative error scores on the WCST, suggesting a possible link between dysfunctions in attention-related networks and impaired attentional control. In contrast, changes in structural connectivity were more limited and not associated with cognitive performance, suggesting that large-scale structural measures may be less sensitive to detect subtle white matter disruptions. Microstructural measures such as FA may offer greater sensitivity, demonstrated by the significant positive correlations between FA in selected white matter pathways and non-perseverative error scores in the mTBI group at the follow-up assessment. Notably, this correlation was absent at the initial assessment, suggesting that the relationship between white matter integrity and cognitive function may not be consistent across time points following mTBI. Overall, these findings highlight the differential impact of mTBI on functional and structural networks, offering insights into the neural mechanisms underlying mTBI-related cognitive deficits.

### Clinical and cognitive changes following mTBI

Individuals with mTBI showed considerable improvements in self-reported outcomes, including the BDI, GOSE, RPCSQ and EQ-5D at the 3-month follow-up, indicating general recovery from post-injury symptoms. However, no significant improvements were observed in K-MoCA or FAB, and performance remained impaired relative to that of controls, suggesting incomplete cognitive recovery. Performance on WCST and DTs provided further insights into the cognitive domains affected by mTBI and their recovery trajectories. Specifically, linear mixed-effects modelling revealed considerable group effects in working memory but not in overall executive functioning or attentional control, highlighting the selective vulnerability of working memory processes following mTBI. However, these effects did not survive correction for multiple comparisons. Taken together, our findings suggest that objective cognitive deficits, particularly in working memory, may persist despite improvements in self-reported symptoms.^47^ This emphasizes the importance of continued cognitive assessment and rehabilitation in patients with mTBI, even after symptomatic recovery. Comprehensive neuropsychological evaluations may be necessary to inform recovery strategies, as self-reported improvements may not fully reflect underlying cognitive impairments.

### Early functional connectivity changes in attentional networks

After correction for multiple comparisons, the only significant effect was a time-related decrease in connectivity between the right DMN-B and right VAN-A, driven by enhanced connectivity in the control group at the baseline assessment compared to the follow-up assessment. No group × time interaction survived correction; therefore, the functional significance of this decrease remains uncertain, and was not interpreted as reflecting injury-related mechanisms. This pattern may instead represent normative fluctuations or non-specific time-related effects. In addition to this statistically corrected finding, several uncorrected patterns provide exploratory insights that may inform future research. Individuals with mTBI exhibited reduced FNC during the baseline phase, particularly in connections between DMN and VAN. While these findings should be interpreted cautiously, as they did not survive multiple comparison correction, they may reflect early alterations in the processing of exogenous stimuli, which could make individuals to be more reactive to external inputs. This interpretation is consistent with previous reports of increased sensitivity to light, noise^48^ and threat-related stimuli following brain injury.^49^ In contrast, reduced connectivity between DMN and DAN may be associated with impaired endogenous, goal-directed attention.^50^ These exploratory findings may have potential clinical relevance, as early FNC reductions could aid in identifying individuals who may benefit from targeted cognitive rehabilitation. Interventions such as attention regulation training could be tailored to address specific attentional deficits and enhance cognitive control following brain injury.^51^

### Task-specific associations between functional network connectivity and cognitive performance

To further investigate the functional relevance of reduced FNC, its relationship with cognitive performance was examined using linear mixed-effects models to determine whether the cognitive relevance of these connections differed between the two time points. Among the 18 FNC pairs that previously showed significant uncorrected main effects of group, 3 DMN-C–DAN connections and left DMN-B and right VAN-B connections showed significant FNC × time interactions for non-perseverative error scores from the WCST. These findings indicated that the strength of the FNC-cognitive association was not consistent across time and varied between the initial and follow-up phases in the mTBI group.

To further clarify whether these time-dependent effects differed between groups, we conducted additional linear mixed-effect analyses considering the identified tracts and found significant group × FNC × time interactions for the connection between left DMN-C and DAN-B (*P* = 0.028), along with a marginally significant group × time interaction (*P* = 0.051). Complementary correlation analyses further illustrated that lower DMN-C connectivity at the baseline phase was associated with poorer attentional performance, whereas this association was not evident at the follow-up phase.

Taken together, the overall pattern highlights a dynamic interplay between DMN-C and the dorsal attention system, suggesting that the coordination between internally oriented processing and goal-directed attentional control is particularly vulnerable and behaviourally consequential during the early stages of mTBI. This coupling was diminished by the follow-up assessment, consistent with the absence of significant group differences in FNC and the loss of simple correlations with cognitive performance. The convergence of reduced early connectivity, significant FNC × time interactions, and the disappearance of positive correlation suggests that DMN–DAN coupling may be particularly relevant to attentional control during the early post-injury period (<1 month), when functional communication between networks is most perturbed.^52,53^ Rather than indicating recovery, these findings imply that the functional relevance of DMN–DAN connectivity to task performance may be temporally constrained, exerting its strongest influence when the system is in a more vulnerable state. Notably, the DMN-C includes the parahippocampal area, a key component of the medial temporal lobe memory system,^54^ and its observed connectivity with attention networks may indicate that memory and attentional control jointly contribute to efficient task execution.

In contrast, backward digit-span scores showed no meaningful association with FNC. Neither the mixed-effects analysis nor the simple correlations revealed a consistent pattern linking working-memory performance to DMN-attention network interactions relevant for WCST performance. This lack of association may indicate a potential dissociation between the neural mechanisms underlying attentional control and those supporting working memory in individuals with mTBI. These findings highlight the importance of task-specific interpretations when relating functional connectivity to cognitive outcomes. Alternatively, working memory performance may depend more heavily on local neural circuits, such as those within the parahippocampal or medial temporal regions, rather than on large-scale attentional networks.

### Subtle changes in structural connectivity

Compared to the changes observed in FNC, those in structural connectivity were more limited in scope and did not remain significant after multiple comparison correction. Although axonal injury or dysfunction is a hallmark of mTBI, the absence of substantial structural connectivity changes suggests subtle or localized microstructural damage^55^ that may spare the long-range pathways connecting DMN, DAN and VAN. This finding aligns with a previous study reporting no major structural connectivity alterations at 1- and 6-month follow-ups.^56^ Structural connectivity is typically inferred from the number of reconstructed white matter streamlines; therefore, substantial disruptions may only become more apparent in cases of more severe or widespread injury.

### White matter microstructure and cognitive performance in mTBI

Large-scale brain networks such as DMN, DAN and VAN are supported by specific white matter tracts that serve as the anatomical basis for interregional communication. These tracts are critically involved in processes including attention, memory and self-reference. Key fibre pathways contributing to these networks include the inferior longitudinal fasciculus, inferior fronto-occipital fasciculus, superior longitudinal fasciculus, forceps minor and anterior thalamic radiation. Assessing the integrity of these tracts using metrics such as FA, AD, RD and MD is essential for understanding how structural abnormalities contribute to cognitive impairments in mTBI.

To directly evaluate these relationships, linear mixed-effects models were used to test whether diffusion metrics predicted cognitive performance across groups and time points. Several pathways showed significant main effects of FA for non-perseverative error scores, and all pathways demonstrated significant main effects of AD, RD and MD after multiple comparison correction, indicating robust associations between microstructural integrity, particularly lower diffusivity and higher anisotropy, and attentional control. However, neither the main effect of time nor any diffusion metric × time interaction reached significance, suggesting that while diffusion properties were associated with behaviour, the strength of these associations did not statistically differ across assessments or between groups.

Nevertheless, several uncorrected diffusion metrics × time interactions were observed for connections between left DAN-B and bilateral DMN-C across FA, RD and MD. Considering that mTBI frequently involves subtle and heterogeneous microstructural alterations,^57^ effects of interest may fall below conservative statistical thresholds in longitudinal designs with modest sample sizes. Therefore, these uncorrected findings should be viewed as exploratory but may nonetheless point to biologically meaningful patterns, warranting further investigation. Additional mixed-effects analyses identified significant group × FA × time interactions for the connection between left DAN-B and bilateral DMN-C (*P* = 0.025 for left; *P* = 0.038 for right) and significant group × time interactions (*P* = 0.028 for left; *P* = 0.040 for right). These exploratory findings indicate that the strength of structure–behaviour relationships may not be static and may shift across the post-injury period. This interpretation aligns with the correlation patterns observed across groups and time points. FA showed a strong positive association with WCST non-perseverative error scores in controls at baseline, consistent with the well-established link between higher anisotropy and better attentional performance.^21,22^ In contrast, this structure–behaviour coupling was absent in the mTBI group within 1-month post-injury. While literature on acute mTBI reports inconsistent findings regarding FA–cognition coupling,^58^ our findings align with studies showing non-significant FA–cognition correlations during the early post-injury phase.^59,60^ However, the pattern reversed upon follow-up: FA–WCST correlations became significantly positive in the mTBI group, whereas the association diminished in controls. This shift mirrors the direction of the uncorrected diffusion metric × time interactions and may suggest that structure–behaviour coupling becomes more detectable in the mTBI group as both cognitive performance and microstructural measures stabilize over time. The absence of coupling at baseline may reflect greater inter-individual variability or transient disruption of the white matter–behaviour relationship during the early phases after mTBI, whereas the follow-up associations may become more detectable as both behavioural performance and diffusion metrics stabilize over time. Conversely, the reduced correlation in controls may reflect restricted variability in white matter integrity and task performance, limiting the observable association at the later time point. In addition, other diffusivity metrics, including AD, RD and MD, showed significant negative correlations with non-perseverative error scores. These findings suggest that increased diffusivity, indicative of axonal and myelin damage, may hinder communication between functional brain networks, further contributing to cognitive deficits observed in mTBI.

In contrast to the findings for attentional control on WCST, the associations between diffusion metrics and backward digit-span performance exhibited a distinct pattern. Consistent with the results, none of the diffusion metric × time interactions were significant after multiple comparison correction, indicating that the strength of these associations did not statistically differ across assessments. Nonetheless, several connections showed interaction effects without multiple comparison corrections, and the results from descriptive correlations revealed meaningful group-specific tendencies. No diffusion measure showed a significant correlation with digit-span performance at either time point in the control group, suggesting that working memory in healthy adults is relatively stable and minimally influenced by variability in white matter microstructure. In contrast, AD, RD and MD demonstrated consistent negative correlations at both assessments in the mTBI group, and FA showed a positive correlation only at follow-up. Although these descriptive shifts did not reach statistical significance in interaction, they may suggest that working memory performance in mTBI remains dependent on microstructural integrity throughout the subacute period, with anisotropy becoming more behaviourally relevant as white matter abnormalities partially resolve or become functionally compensated. These patterns should be interpreted cautiously but may provide early clues regarding differential recovery trajectories for attentional control and working memory after mild TBI.

Taken together, these findings highlight that diffusion–behaviour coupling may evolve during this window period. While uncorrected interactions should be interpreted cautiously, their anatomical specificity and convergence across diffusion metrics suggest that they may represent meaningful biological signals rather than statistical noise. Larger longitudinal studies are warranted to determine whether these early indications of temporal modulation reflect genuine microstructural reorganization during recovery from mTBI.

### Limitations

Although previous studies have consistently reported an anti-correlation between the DMN and task-positive networks,^61,62^ this pattern was not observed in the present study. This discrepancy may be attributed to the use of predefined networks from the Schaefer functional atlas,^37^ where relatively large regions may have averaged signals across broader areas within each network, reducing the granularity of observed correlation patterns.^63^ Nevertheless, this atlas remains appropriate for the current analysis as it is based on functional connectivity and is well-suited for examining large-scale networks. While considerable disruptions in FNC were observed, changes in structural connectivity were less prominent, possibly because our single-shell DTI acquisition with a moderate number of diffusion directions is inherently limited in resolving complex or crossing-fibre configurations. Such a limitation may reduce sensitivity to detect subtle or localized microstructural damage, particularly in tract-based connectivity estimates. High-angular-resolution diffusion imaging and diffusion spectrum imaging offer better differentiation of complex fibre structures and may provide greater sensitivity to subtle white matter changes.^64,65^ In addition, the 3-month follow-up period may have been too short to fully capture the long-term effects of mTBI. Longer follow-ups are required to assess the trajectory of recovery and persistence of cognitive deficits. This study focused specifically on alterations in attentional networks following mTBI. However, the lack of a more specialized assessment of attention limits the ability to establish a direct link between attention network changes and attentional deficits. In addition, although our analyses were restricted to DAN, VAN and DMN, incorporating additional networks, including the executive control network, is essential to provide a more comprehensive understanding of network-level alterations underlying post-TBI attentional impairments. Finally, the relatively small sample size may have limited the detection of subtle effects. A larger sample would improve statistical power, enable more robust subgroup analyses, and enhance the generalizability of the findings.

## Conclusions

Alterations in FNC were more prominent than those in structural connectivity following mTBI, particularly in VAN, which is associated with exogenous attention. Greater reductions in FNC were linked to poorer attentional control during baseline assessment, although these alterations resolved at the 3-month follow-up. Improved attentional control was associated with increased FA at follow-up, suggesting that ongoing microstructural changes may influence cognitive performance. Although FNC appeared to recover within 3 months, its precise role in cognitive recovery remains unclear. Notably, working memory capacity was not associated with changes in FNC but was related to microstructural integrity at both time points. This dissociation may reflect the reliance of working memory on stable structural connections, whereas functional networks may be more variable and less directly involved in memory processes.

## Supplementary Material

fcag072_Supplementary_Data

## Data Availability

The data that support the findings of this study are available from the corresponding author upon reasonable request. Code for statistical analyses can be found at https://github.com/Eun517/mtbi-structure-function-longitudinal.
