# Supplementary material for: Time course of functional and structural brain network changes after mild traumatic brain injury
Source: Brain Commun. 2026 Mar 16;8(2):fcag072. doi: 10.1093/braincomms/fcag072 (PMC13009404; doi:10.1093/braincomms/fcag072)
Supplement: fcag072_Supplementary_Data [file fcag072_supplementary_data.docx]

**Supplementary Materials**

**Title: Time course of functional and structural brain network changes after mild traumatic brain injury**

**Authors:** Eunkyung Kim,^1,2^ Han Gil Seo,^1,3^ Roh-Eul Yoo^4^ and Byung-Mo Oh^1,3,5,*^

**Affiliation:**

^1^ Department of Rehabilitation Medicine, Seoul National University Hospital, Seoul, Korea

^2^ Biomedical Research Institute, Seoul National University Hospital, Seoul, Korea

^3^ Department of Rehabilitation Medicine, Seoul National University College of Medicine, Seoul, Korea

^4^ Department of Radiology, Seoul National University College of Medicine and Seoul National University Hospital, Seoul, Korea

^5^ Institute on Aging, Seoul National University, Seoul, Korea

Correspondence to:

Byung-Mo Oh, MD, PhD, Department of Rehabilitation Medicine, Seoul National University Hospital and Seoul National University College of Medicine, Seoul, Republic of Korea ([moya1@snu.ac.kr](mailto:moya1@snu.ac.kr))

Correspondence may also be sent to:

Eunkyung Kim, PhD, Department of Rehabilitation Medicine and Biomedical Research Institute, Seoul National University Hospital, Seoul, Republic of Korea (eunkyung@snu.ac.kr)

**Supplementary Results**

**1. Demographic data**

Among the 55 enrolled patients with mild traumatic brain injury (mTBI), four withdrew before their first visit. Five completed the initial assessments and brain imaging but declined further participation. Four patients were excluded owing to abnormal brain findings, including subependymal gray matter heterotopia, a small hemorrhagic lesion, nodular lesions (raising suspicion of cavernous malformation), and an 8 mm calcified lesion possibly representing a meningioma or vascular calcification. One individual with mTBI was also excluded owing to low-quality diffusion tensor imaging data. Ultimately, 41 individuals with mTBI (mean age at the time of first magnetic resonance imaging [MRI]: 48.7 ± 15.8 years, 22 female) were included in the analyses. They underwent their first MRI scan within one-month post-injury (20.0 ± 6.3 days) and a second scan three months later (102.5 ± 17.4 days), with an average interval of 82.4 ± 17.8 days between scans. Clinical assessments were conducted within one week of each imaging session.

In the control group, 53 participants were initially enrolled, but 18 were excluded: two withdrew before their first visit, three did not return for follow-up, five had scores <23 on the Korean version of the Montreal Cognitive Assessment, and six had abnormal brain findings, including a 5 mm cerebrospinal fluid-filled lesion, cavernous angioma, microbleeds in the right centrum semiovale and left parietal lobe, multiple cerebral microbleeds, and a calcified meningioma. One participant did not complete the cognitive test owing to technical issues. Another was excluded because their matched patient was excluded because of poor data quality. The remaining 35 healthy controls (44.6 ± 12.8 years at the time of first imaging) underwent both first and second brain imaging sessions with intervals similar to their matched patients with mTBI (81.1 ± 13.2 days). The scan intervals were generally consistent, with differences of approximately two weeks for most matched pairs. However, one participant had a longer interval (15 days) due to scheduling difficulties, and another had a shorter interval (66 days) due to a temporary loss of contact with their matched patient. Six older patients aged 60, 66, 71, 76, and 84 years did not have matched controls.

To confirm that loss to follow-up did not bias the longitudinal analyses, baseline demographic and clinical variables, including age, sex, BDI, FAB, K-MoCA, GOSE, RPCSQ, and EQ-5D, were compared between participants who completed both visits and those who did not, using the Wilcoxon rank-sum test (*p* < 0.05) and Fisher’s exact test (*p* < 0.05) as appropriate. These analyses revealed no significant group differences, indicating that loss to follow-up was unlikely to bias the results.

**2. Changes in functional network connectivity**

Linear mixed-effects models revealed significant main effects of the group in 18 pairs of functional network connectivity (FNC) (Figure 2, Table 3) involving the default mode network (DMN), ventral attention network (VAN), and dorsal attention network (DAN). However, no group differences remained statistically significant after correction for multiple comparisons. Specifically, significant group effects were observed in FNC between the left DMN-A and bilateral VAN-B; left DMN-B and bilateral VAN-B; and left DMN-C and bilateral VAN-A, left DAN-A, and left DAN-B. In the right hemisphere, significant group effects were found between the right DMN-A and both left VAN-A and bilateral VAN-B, and between the right DMN-B and right VAN-B. Additional group effects were observed between the right DMN-C and bilateral VAN-A, bilateral VAN-B, and left DAN-A and DAN-B. Post hoc analyses showed that FNC was lower in the mTBI group than in the controls at the initial time point, with no significant group differences observed at follow-up.

Significant main effects of time were found in six FNC pairs: left DMN-B with bilateral VAN-B; bilateral DMN-B with right VAN-B; right DMN-A with right VAN-B; and right DMN-B with bilateral VAN-B (Table 3). After correcting for multiple comparisons, only the FNC between the right DMN-B and right VAN-B remained significant. Post-hoc analyses revealed that in the control group, FNC decreased from initial to follow-up assessments in five pairs, whereas FNC between the bilateral DMN-B increased over time. In contrast, the mTBI group showed no significant time-dependent changes in FNC.

| **Supplementary Table 1 Anatomical regions and parcel indices from the Schaefer 400-parcel atlas comprising each subnetwork of the DAN, VAN, and DMN** | | | | |
| --- | --- | --- | --- | --- |
| **Subnetwork** | **Left hemisphere** | | **Right hemisphere** | |
|  | **Region names** | **Schaefer labels** | **Region names** | **Schaefer labels** |
| **DAN-A** |  |  |  |  |
|  | Temporal-Occipital Cortex | 60-63 | Temporal-Occipital Cortex | 259-261 |
|  | Parieto-Occipital Cortex | 64, 65 | Parieto-Occipital Cortex | 262-264 |
|  | Superior Parietal Lobule | 66-72 | Superior Parietal Lobule | 265-272 |
| **DAN-B** |  |  |  |  |
|  | Postcentral Cortex | 73-81 | Temporal-Occipital Cortex | 273 |
|  | Frontal Eye Fields | 82-84 | Postcentral Cortex | 274-281 |
|  | Ventral Precentral Cortex | 85 | Frontal Eye Fields | 282-284 |
| **VAN-A** |  |  |  |  |
|  | Parietal Operculum | 86-88 | Parietal Operculum | 285-287 |
|  | Insula | 89-92 | Precentral Cortex | 288 |
|  | Frontal Operculum | 93, 94 | Insula | 289-292 |
|  | Parietal Medial Cortex | 95-97 | Frontal Operculum | 293-295 |
|  | Frontal Medial Cortex | 98-100 | Frontal Medial Cortex | 296, 299, 302, 303 |
|  |  |  | Parietal Medial Cortex | 297, 298, 300, 301 |
| **VAN-B** |  |  |  |  |
|  | Lateral Prefrontal Cortex | 101-103 | Inferior Parietal Lobule | 304 |
|  | Insula | 104-106 | Lateral Ventral Prefrontal Cortex | 305 |
|  | Orbitofrontal Cortex | 107 | Lateral Prefrontal Cortex | 306-308 |
|  | Medial Posterior Prefrontal Cortex | 108 | Insula | 309, 310 |
|  |  |  | Medial Posterior Prefrontal Cortex | 311, 312 |
| **DMN-A** |  |  |  |  |
|  | Inferior Parietal Lobule | 149, 150 | Temporal Cortex | 358 |
|  | Dorsal Prefrontal Cortex | 151-153 | Inferior Parietal Lobule | 359, 360 |
|  | Precuneus Posterior Cingulate Cortex | 154-160 | Dorsal Prefrontal Cortex | 361, 362 |
|  | Medial Prefrontal Cortex | 161-166 | Precuneus Posterior Cingulate Cortex | 363-367 |
|  |  |  | Medial Prefrontal Cortex | 368-373 |
| **DMN-B** |  |  |  |  |
|  | Temporal Cortex | 167-172 | Temporal Cortex | 374, 375 |
|  | Inferior Parietal Lobule | 173, 174 | Anterior Temporal Cortex | 376 |
|  | Dorsal Prefrontal Cortex | 175-180 | Dorsal Prefrontal Cortex | 377-381 |
|  | Lateral Prefrontal Cortex | 181, 182 | Ventral Prefrontal Cortex | 382-384 |
|  | Ventral Prefrontal Cortex | 183-187 |  |  |
| **DMN-C** |  |  |  |  |
|  | Inferior Parietal Lobule | 188 | Inferior Parietal Lobule | 385, 386 |
|  | Retrosplenial Cortex | 189-191 | Retrosplenial Cortex | 387, 388 |
|  | Parahippocampal Cortex | 192-194 | Parahippocampal Cortex | 389, 390 |
| **Abbreviations**: DAN-A, dorsal attention network A; DAN-B, dorsal attention network B; VAN-A, ventral attention network A; VAN-B, ventral attention network B; DMN-A, default mode network A; DMN-B, default mode network B; DMN-C, default mode network C. | | | | |

| **Supplementary Table 2 Summary data and post-hoc analyses of computerized neurocognitive function test within and between groups** | | | | | | | | | | |
| --- | --- | --- | --- | --- | --- | --- | --- | --- | --- | --- |
|  | mTBI | | | | Controls | | | | mTBI vs. Controls | |
|  | Baseline | Follow-up | *p-*value | *z-*value | Baseline | Follow-up | *p-*value | *z-*value | Baseline (*p-*value) | Follow-up (*p-*value) |
| Card sorting^a^ |  |  |  |  |  |  |  |  |  |  |
| Total error | 52.82 (9.25) | 55.66 (11.45) | 0.162 | -1.40 | 55.69 (11.10) | 54.69 (8.93) | 0.511 | 0.66 | 0.456 | 0.746 |
| Perseverative Response | 56.92 (10.49) | 59.79 (12.11) | 0.174 | -1.36 | 57.74 (11.75) | 58.46 (10.57) | 0.820 | -0.23 | 0.939 | 0.975 |
| Perseverative Errors | 54.92 (8.25) | 57.42 (9.87) | 0.203 | -1.27 | 55.77 (10.09) | 56.71 (9.35) | 0.608 | -0.51 | 0.900 | 0.929 |
| Non-Perseverative Errors | 53.24 (9.48) | 55.21 (10.82) | 0.313 | -1.01 | 56.43 (12.53) | 55.20 (11.69) | 0.603 | 0.52 | 0.541 | 0.789 |
| Digit span |  |  |  |  |  |  |  |  |  |  |
| Forward | 49.93 (15.49) | 53.20 (18.08) | 0.198 | -1.29 | 56.31 (16.54) | 59.80 (17.38) | 0.152 | -1.43 | 0.086 | 0.088 |
| Backward | 51.46 (12.22) | 53.22 (14.68) | 0.478 | -0.71 | 58.34 (12.26) | 60.43 (13.49) | 0.234 | -1.19 | 0.015 | 0.023 |

^a^Three individuals with mild traumatic brain injury had missing initial data for the Wisconsin Card Sorting Test and were excluded from the statistical analysis for this subtest.

**Abbreviations**: mTBI, mild traumatic brain injury.

| **Supplementary Table 3 Results of the linear mixed-effects analysis comparing structural connectivity between individuals with mTBI and healthy controls** | | | | | | | | | |
| --- | --- | --- | --- | --- | --- | --- | --- | --- | --- |
| Structural network | | Main effects (linear mixed-effects) | | | | Post-hoc comparisons (*p*-value) | | | |
|  |  | Group | | Time | | mTBI vs. Controls | | Baseline vs. Follow-up | |
|  |  | β | *p*-value | β | *p*-value | Baseline | Follow-up | mTBI | Controls |
| L. DAN-A | L. DMN-B | 0.002 | 0.359 | 0.004 | *0.042* | 0.421 | 0.693 | 0.419 | 0.120 |
|  | R. VAN-A | -0.0002 | 0.617 | 0.0004 | *0.024* | 0.650 | 0.214 | 0.547 | 0.026 |
| L. DAN-B | L. DAN-A | -0.011 | *0.046* | -0.003 | 0.137 | 0.057 | 0.387 | 0.134 | 0.208 |
|  | R. VAN-A | -0.002 | *0.016* | 0.0003 | 0.576 | 0.019 | 0.017 | 0.485 | 0.605 |
| **Abbreviations:** L., left; R., right; DAN-A, dorsal attention network A; DAN-B, dorsal attention network B; DMN-B, default mode network B; VAN-A, ventral attention network A; mTBI, mild traumatic brain injury. | | | | | | | | | |

| **Supplementary Table 4 Linear mixed-effects results (*p*-values) for association between diffusion metrics and non-perseverative error scores in the mTBI group** | | | | | | | | | | | | | |
| --- | --- | --- | --- | --- | --- | --- | --- | --- | --- | --- | --- | --- | --- |
| Functional network connectivity | | FA | | | AD | | | RD | | | MD | | |
|  |  | FA | Time | FA × Time | AD | Time | AD × Time | RD | Time | RD × Time | MD | Time | MD × Time |
| L. DMN-A | L. VAN-B | 0.107 | 0.101 | 0.059 | 0.003^*^ | 0.551 | 0.565 | 0.004^*^ | 0.299 | 0.319 | 0.003^*^ | 0.382 | 0.399 |
|  | R. VAN-B | 0.021^*^ | 0.268 | 0.283 | 0.019^*^ | 0.393 | 0.406 | 0.014^*^ | 0.264 | 0.283 | 0.015^*^ | 0.302 | 0.319 |
| L. DMN-B | L. VAN-B | 0.028^*^ | 0.190 | 0.260 | 0.017^*^ | 0.300 | 0.313 | 0.016^*^ | 0.207 | 0.226 | 0.016^*^ | 0.234 | 0.250 |
|  | R. VAN-B | 0.221 | 0.059 | 0.062 | 0.002^*^ | 0.147 | 0.151 | 0.002^*^ | 0.070 | 0.076 | 0.002^*^ | 0.086 | 0.091 |
| L. DMN-C | L. VAN-A | 0.052 | 0.132 | 0.281 | 0.015^*^ | 0.465 | 0.480 | 0.015^*^ | 0.277 | 0.299 | 0.014^*^ | 0.332 | 0.351 |
|  | R. VAN-A | 0.043 | 0.142 | 0.165 | 0.006^*^ | 0.616 | 0.631 | 0.008^*^ | 0.328 | 0.351 | 0.007^*^ | 0.411 | 0.431 |
|  | L. DAN-A | 0.396 | 0.067 | 0.082 | 0.007^*^ | 0.123 | 0.129 | 0.008^*^ | 0.105 | 0.116 | 0.007^*^ | 0.106 | 0.115 |
|  | L. DAN-B | 0.200 | 0.037 | 0.013 | 0.003^*^ | 0.067 | 0.070 | 0.004^*^ | 0.031 | 0.035 | 0.003^*^ | 0.041 | 0.044 |
| R. DMN-A | L. VAN-A | 0.020^*^ | 0.216 | 0.249 | 0.010^*^ | 0.397 | 0.408 | 0.008^*^ | 0.278 | 0.297 | 0.008^*^ | 0.314 | 0.330 |
|  | L. VAN-B | 0.035^*^ | 0.241 | 0.125 | 0.008^*^ | 0.467 | 0.480 | 0.008^*^ | 0.297 | 0.319 | 0.007^*^ | 0.349 | 0.367 |
|  | R. VAN-B | 0.094 | 0.108 | 0.104 | 0.022^*^ | 0.265 | 0.274 | 0.020^*^ | 0.128 | 0.140 | 0.020^*^ | 0.160 | 0.171 |
| R. DMN-B | R. VAN-B | 0.120 | 0.081 | 0.098 | 0.005^*^ | 0.125 | 0.130 | 0.006^*^ | 0.064 | 0.070 | 0.006^*^ | 0.077 | 0.082 |
| R. DMN-C | L. VAN-A | 0.123 | 0.106 | 0.092 | 0.002^*^ | 0.190 | 0.197 | 0.001^*^ | 0.120 | 0.130 | 0.001^*^ | 0.134 | 0.142 |
|  | L. VAN-B | 0.056 | 0.137 | 0.136 | 0.007^*^ | 0.631 | 0.649 | 0.008^*^ | 0.351 | 0.376 | 0.007^*^ | 0.436 | 0.458 |
|  | R. VAN-A | 0.024^*^ | 0.212 | 0.231 | 0.015^*^ | 0.417 | 0.433 | 0.013^*^ | 0.283 | 0.306 | 0.013^*^ | 0.325 | 0.345 |
|  | R. VAN-B | 0.100 | 0.135 | 0.152 | 0.014^*^ | 0.174 | 0.181 | 0.014^*^ | 0.086 | 0.095 | 0.014^*^ | 0.105 | 0.112 |
|  | L. DAN-A | 0.136 | 0.035 | 0.042 | 0.004^*^ | 0.163 | 0.170 | 0.005^*^ | 0.067 | 0.074 | 0.004^*^ | 0.088 | 0.095 |
|  | L. DAN-B | 0.269 | 0.036 | 0.019 | 0.003^*^ | 0.121 | 0.125 | 0.004^*^ | 0.034 | 0.038 | 0.003^*^ | 0.052 | 0.056 |
| ^*^Multiple comparisons were corrected using the false discovery rate method.  **Abbreviations**: mTBI, mild traumatic brain injury; FA, fractional anisotropy; AD, axial diffusivity; RD, radial diffusivity; MD, mean diffusivity; L., left; R., right; DMN-A, default mode network A; DMN-B, default mode network B; DMN-C, default mode network C; VAN-A, ventral attention network A; VAN-B, ventral attention network B; DAN-A, dorsal attention network A; DAN-B, dorsal attention network B. | | | | | | | | | | | | | |
